# Placentation in the paca (*Agouti paca *L)

**DOI:** 10.1186/1477-7827-3-9

**Published:** 2005-02-28

**Authors:** Marina Bonatelli, Anthony M Carter, Marcia R Fernandes Machado, Moacir F De Oliveira, Marcelo Cardoso de Lima, Maria Angelica Miglino

**Affiliations:** 1 Department of Surgery, School of Veterinary Medicine, University of Sao Paulo, Sao Paulo, Brazil; 2 Department of Physiology and Pharmacology, University of Southern Denmark, Odense, Denmark; 3 Paulista State University, Jaboticabal, Sao Paulo, Brazil; 4 Mossoró Superior School of Agriculture, Rio Grande do Norte, Brazil

## Abstract

**Background:**

The paca is a South American rodent with potential as a commercial food animal. We examined paca placenta as part of a wider effort to understand the reproductive biology of this species.

**Methods:**

Thirteen specimens between midgestation and term of pregnancy were studied by light and transmission electron microscopy.

**Results:**

The placenta is divided into several lobes separated by interlobular trophoblast. Maternal arterial channels and fetal veins are found at the centre of each lobe. In the labyrinth, maternal blood flows through trophoblast-lined lacunae in close proximity to the fetal capillaries. The interhaemal barrier is of the haemomonochorial type with a single layer of syncytiotrophoblast. Caveolae occur in the apical membrane of the syncytiotrophoblast and recesses in the basal membrane, but there is no evidence of transtrophoblastic channels. The interlobular areas consist of cords of syncytiotrophoblast defining maternal blood channels that drain the labyrinth. Yolk sac endoderm covers much of the fetal surface of the placenta. The subplacenta comprises cytotrophoblast and syncytiotrophoblast. There are dilated intercellular spaces between the cytotrophoblasts and lacunae lined by syncytiotrophoblast. In the junctional zone between subplacenta and decidua, there are nests of multinucleated giant cells with vacuolated cytoplasm. The entire placenta rests on a pedicle of maternal tissue. An inverted yolk sac placenta is also present. The presence of small vesicles and tubules in the apical membrane of the yolk sac endoderm and larger vesicles in the supranuclear region suggest that the yolk sac placenta participates in maternal-fetal transfer of protein.

**Conclusion:**

The paca placenta closely resembles that of other hystricomorph rodents. The lobulated structure allows for a larger exchange area and the development of precocial young.

## Introduction

The paca (*Agouti paca*, L) is a South American rodent that lives in forested habitats near water and feeds largely on fallen fruit. It is hunted for its meat, which is considered a delicacy, and is an important source of animal protein for rural populations. This has led to indiscriminate exploitation, resulting in a significant reduction in the population density of this species in Brazil.

We here describe the morphology of paca placenta as revealed by light microscopy and transmission electron microscopy. This study is part of a wider effort to document the reproductive physiology of paca. It is hoped that the information obtained will contribute to a rational strategy for conservation of the species and possibly for production, as paca has great potential as a commercial food animal [1].

## Materials and methods

The observations are based on placentae collected from 13 pregnant females. One was in early gestation, two in midgestation and nine near term of pregnancy. This material was collected at Paulista State University, Jaboticabal, Sao Paulo, Brazil. The research was authorized by the Brazilian Institute of the Environment and Renewable Natural Resources (IBAMA). The experimental protocol was approved by the Bioethics Committee of the School of Veterinary Medicine, University of Sao Paulo.

The animals were sedated with azaperone (Stresnil, Janssen Pharmaceutica, Brazil; 0.1 mg/kg I.M.) and given atropine (0.5 mg I.M.). Anaesthesia was induced with xylazine (Coopazine, Coopers Brazil, Sao Paulo, S.P., Brazil; 1.5 mg/kg I.M.) and ketamine (Holliday Scott S.A., Brazil; 20 mg/kg I.M.). Hemihysterectomy was then performed under aseptic conditions during inhalation anaesthesia with halothane (Hoechst, Frankfurt, Germany). Postoperatively the animals were treated with benzyl penicillin and streptomycin (Pentabiotico^®^, Fort Dodge, Campinas, S.P., Brazil; 8,000–24,000 IU/kg I.M.) and an analgetic (buprenorphine, Temgesic^®^, Schering-Plough, S.P., Brazil). A detailed description of the anaesthesia and surgical procedures has been published elsewhere [2].

Pieces of ten placentae were fixed in Bouin's solution or 10% formaldehyde and processed by standard histological procedures for embedding in paraplast, then sectioned at 7 μm (automatic microtome, Model RM2155, Leica, Germany). Sections were stained with haematoxylin and eosin, Masson's trichrome or Gomori's trichrome or by the periodic acid Schiff (PAS) reaction with haematoxylin as counterstain.

Seven placentae were processed for transmission electron microscopy. Small samples were fixed in 2.5% glutaraldehyede in 0.1 M phosphate buffer, pH 7.4, washed in buffer and post-fixed in 1% osmium tetroxide (Polysciences, Warrington, PA, USA). They were then dehydrated, washed with propylene oxide and embedded in Spurr's resin (Spurr's Kit, Electron Microscopy Sciences, CO, U.S.A.). 60 nm sections were made and stained with 2% uranyl acetate (5 minutes) and 0.5% lead citrate (10 minutes). The ultrastructural observations were made with a transmission electron microscope (JEOL 1010, Peabody, MA, U.S.A).

## Results

**Figure 1:**
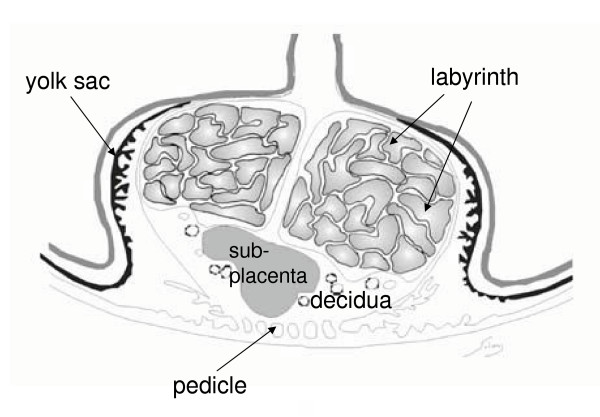
Schematic drawing of the paca placenta. The labyrinth is divided into lobes separated by interlobular trophoblast. Beneath it is found the subplacenta and then the decidua. There is a tenuous attachment to the uterine wall, the pedicle or mesoplacenta. An inverted yolk sac placenta is present throughout gestation.

The overall plan of the paca placenta is shown schematically in Figure 1. The labyrinth is divided into lobes separated by interlobular trophoblast. Beneath this is the subplacenta, a structure unique to hystricomorph rodents. The junctional zone between these structures and the maternal decidua contains nests of giant cells. The placenta is attached to the uterus by a pedicle of maternal tissue. In addition, there is an inverted yolk sac placenta, which connects with the fetal surface of the chorioallantoic placenta.

### Histology

**Figure 2:**
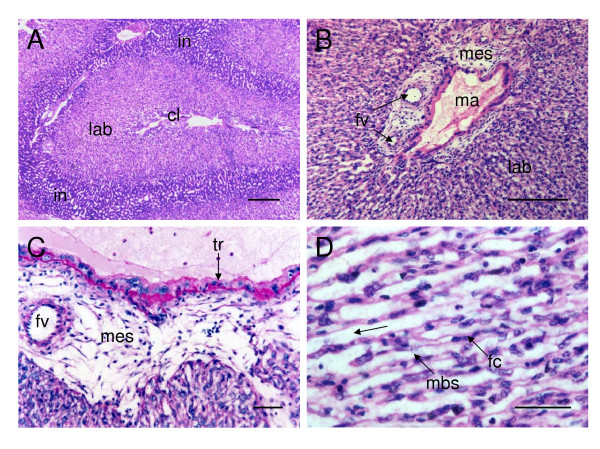
The labyrinth of the paca placenta. (A) The central region of a lobe (cl), a labyrinthine region (lab) and interlobular regions (in). Haematoxylin and eosin. (B) Central region of a placental lobe, showing the presence of fetal veins (fv), maternal arterial blood spaces (ma) lined by trophoblast cells, and the mesenchyme (mes) surrounding these structures. Haematoxylin and eosin. (C) Detail to show the trophoblastic lining of maternal blood spaces (tr) and the intact walls of small fetal veins. PAS. (D) The placental labyrinth, showing the radial disposition (←) of the trophoblastic columns, the fetal capillaries (fc) and maternal blood spaces (mbs). Haematoxylin and eosin. Scale bars: 500 μm (A), 200 μm (B); 50 μm (C, D).

The centres of the lobes, the labyrinth and the interlobular regions are clearly defined (Figure 2A). The central region of each lobe contains fetal and maternal vessels around which there is a considerable quantity of connective tissue (fetal mesenchyme; Figure 2B). The vessels carrying maternal arterial blood lack endothelium and they are lined by trophoblast cells (Figure 2C).

The most extensive portion of the lobe is the labyrinth. Due to close proximity between maternal and fetal blood vessels, it is the region where most maternal-fetal exchange takes place. The maternal blood spaces or lacunae are not lined by endothelium; they are defined by trophoblastic cell columns or cords. The cell columns are radially arranged as is apparent when the lobe is seen in cross section (Figure 2D). The syncytial nature of the trophoblastic columns is indicated by the close proximity of their nuclei.

**Figure 3:**
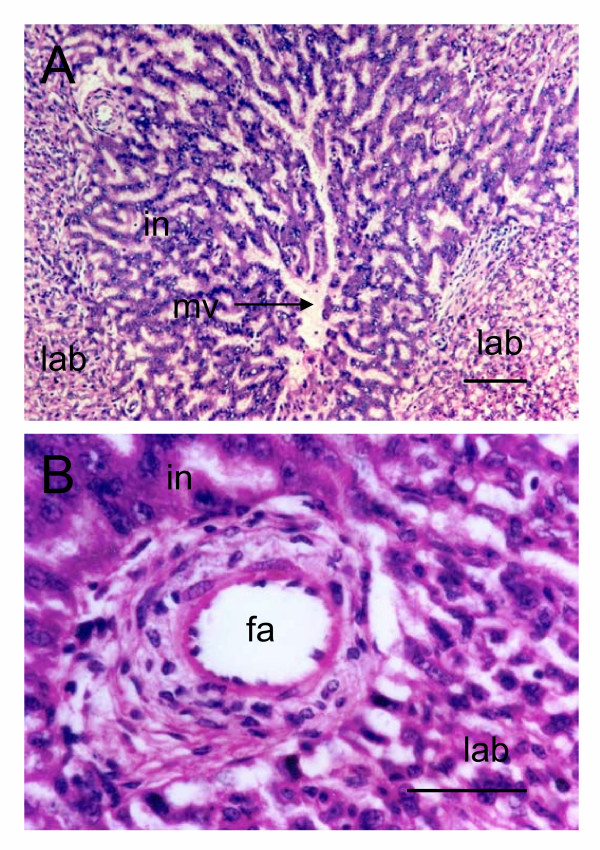
The interlobium of the paca placenta. (A) Channels in the interlobium (in) drain the maternal blood spaces of the labyrinth (lab) and converge on larger venous blood spaces (mv). Haematoxylin and eosin. (B) Fetal artery (fa) at the border between an interlobular region and the labyrinth at the periphery of a lobe. Haematoxylin and eosin. Scale bars: 100 μm (A); 50 μm (B).

The interlobular regions comprise cords of syncytiotrophoblast with abundant basophilic cytoplasm. These cords define maternal blood channels. The channels converge upon larger blood spaces, still without an endothelial lining, that receive blood from two or more adjacent lobes. Thus, each interlobular region is common to several lobes (Figure 3A). The interlobular regions also contain fetal arteries (Figure 3B) that give rise to the capillaries of the labyrinth.

### Placental surface

**Figure 4:**
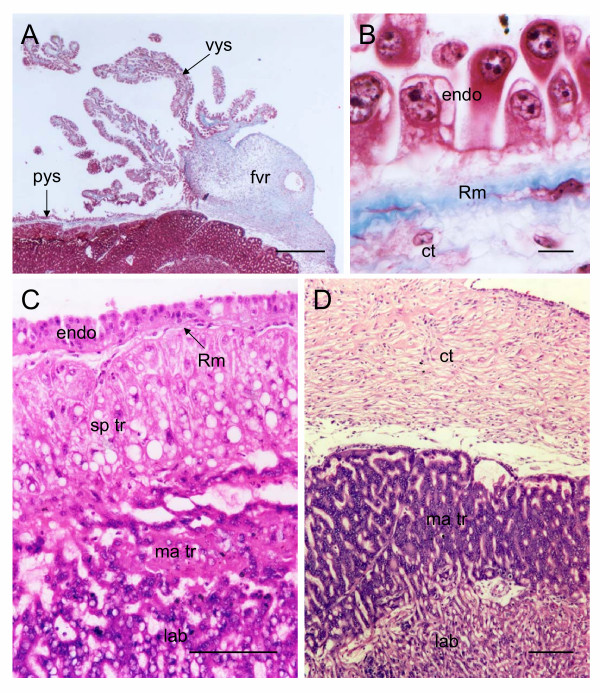
Fetal surface of the paca placenta. (A) The visceral yolk sac (vys) attaches to the surface of the chorioallantoic placenta. Just before attachment it forms the fibrovascular ring (fvr). Left of the point of attachment, the endoderm continues as the parietal yolk sac (pys). To the right, the surface is covered by connective tissue. Masson's trichrome. (B) The parietal yolk sac endoderm (endo) forms a layer of epithelial cells that rests on Reichert's membrane (Rm). Immediately below the membrane are scattered connective tissue cells (ct). Masson's trichrome. (C) Beneath the endoderm and Reichert's membrane are spongiotrophoblast cells (sp tr), with a vacuolated appearance, marginal syncytium (ma tr) and a portion of the labyrinth (lab). Haematoxylin and eosin. (D) The centre of the placental disk is covered by connective tissue. Beneath this are marginal trophoblast and labyrinth. Haematoxylin and eosin. Scale bars: 500 μm (A); 10 μm (B); 100 μm (C-D).

Most of the surface of the placental disk is covered by an epithelium formed by the endoderm of the parietal yolk sac (Figure 4A–C). An almost continuous Reichert's membrane can be demonstrated (Figure 4B). Beneath it is a layer of spongiotrophoblast. These cells differ from those that occur in the marginal and interlobular trophoblast. They are larger in size and have a rounded and vacuolated appearance (Figure 4C). The centre of the fetal surface of the placenta, including the attachment of the cord, is covered by a layer of connective tissue (Figure 4D).

### Subplacenta, junctional zone and pedicle

**Figure 5:**
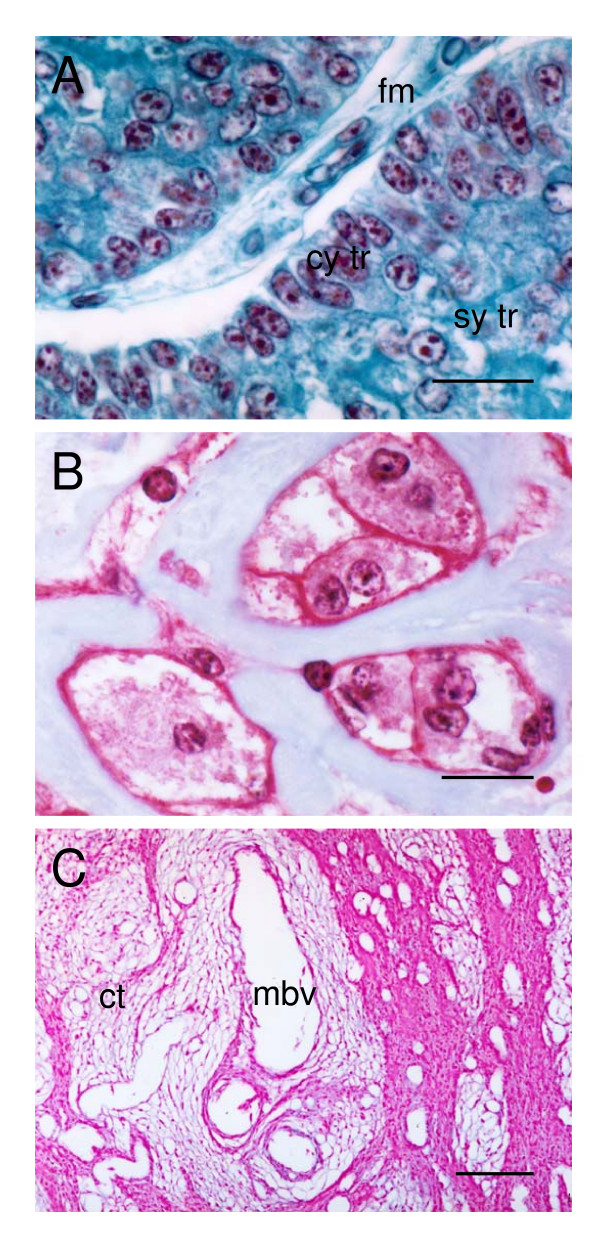
Subplacenta, junctional zone and pedicle of the paca placenta. (A) Subplacenta. A layer of cytotrophoblasts (cy tr) is supported on lamellae of fetal mesenchyme (fm). Beneath it is the subplacental syncytiotrophoblast (sy tr). Gomori's trichrome. (B) Groups of multinucleated giant cells with a finely granular cytoplasm are found between the subplacenta and decidua. They are bordered by connective tissue. Masson's trichrome. (C) Middle portion of the placental pedicle, showing a large number of maternal blood vessels (mbv) and connective tissue fibers (ct). Haematoxylin and eosin. Scale bars: 20 μm (A-B); 200 μm (C).

The subplacenta is organized as folded lamellae of cytotrophoblasts supported on a thin layer of mesenchyme carrying fetal vessels (Figure 5A). The cytotrophoblast is multilaminar and mitotic figures are common here in early and midgestation. Beneath this layer is syncytiotrophoblast, which contains vacuoles and PAS-positive material. In late gestation this region becomes more compact and the entire subplacenta undergoes a process of degeneration.

Groups of giant cells are found in the junctional zone between the subplacenta and decidua (Figure 5B). They are multinucleated and have a finely granular, basophilic cytoplasm. They are PAS-positive.

The placenta is attached to the uterus by a placental pedicle made up of uterine tissue. In the upper region of this pedicle, a fine and discontinuous layer of connective tissue is interposed between the maternal tissue and fetal trophoblast. In the middle portion of the pedicle, a large number of vessels pass to or from the placenta. This region is characterized by dense fibres of connective tissue externally and of looser connective tissue around the vessels (Figure 5C). A layer of squamous epithelial cells with simple, round nuclei, lightly condensed chromatin and clear cytoplasm covers the entire pedicle (not shown).

### Yolk Sac Placenta

**Figure 6:**
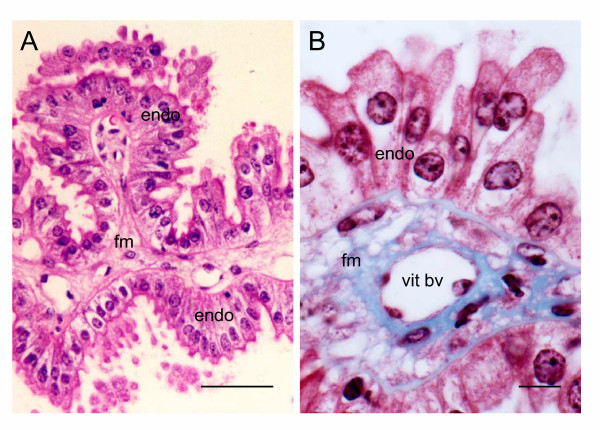
Yolk sac placenta of the paca. (A) The visceral yolk sac is complexly folded with columnar epithelial cells of endodermal origin (endo) supported by fetal mesenchyme (fm). Haematoxylin and eosin. (B) The mesenchyme (stained blue) contains vitelline blood vessels (vit bv). Masson's trichrome. Scale bars: 50 μm (A); 10 μm (B).

There is an inverted yolk sac placenta, which is attached to the fetal surface of the chorioallantoic placenta. Just before attachment it forms the fibrovascular ring (Figure 4A). The yolk sac exhibits numerous digitiform projections (Figure 6A), which sometimes are branched. They consist of a mesenchymal core covered by a layer of endoderm. The latter forms a simple columnar epithelium of cells with apically situated cell nuclei (Figure 6B).

### Ultrastructure

**Figure 7:**
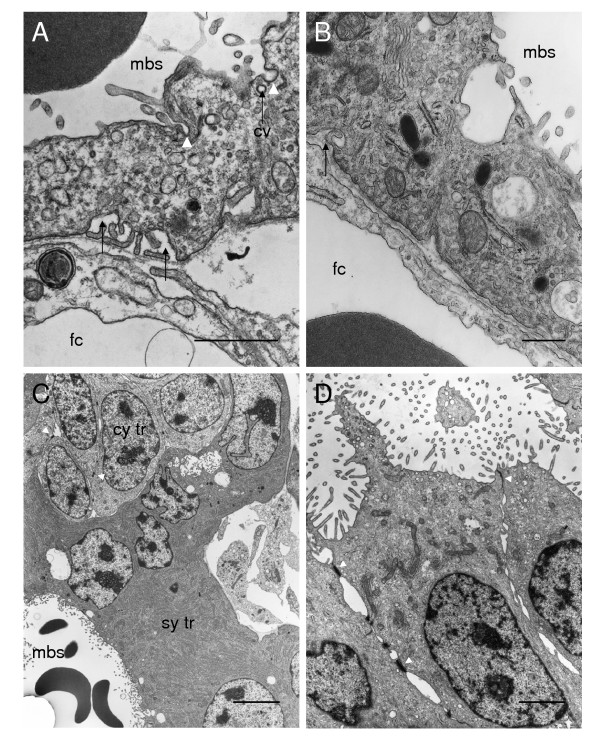
Ultrastructure of the labyrinth, interlobium and parietal yolk sac endoderm. (A-B) The interhaemal barrier. Only a single layer of syncytiotrophoblast is interposed between the blood flowing in the maternal blood spaces (mbs) and the endothelium of the fetal capillary (fc). The apical membrane contains invaginations (arrowheads) and the cytoplasm includes small coated vesicles (cv) and larger vacuoles. There are recesses in the basal membrane (arrows). (C) Interlobium. Most of the trophoblast is syncytial (sy tr) with abundant rough endoplasmatic reticulum. There are many microvilli where it is in contact with the maternal blood space. Cytotrophoblast cells with large nuclei are found in some regions of the interlobium. Desmosomes are found between the lateral membranes of adjacent cells (arrowheads). (D) Parietal yolk sac endoderm. These columnar cells have numerous microvilli at the apical surface. The supranuclear cytoplasm contains vacuoles and vesicles, tubular mitochondria and rough endoplasmic reticulum. Desmosomes are found between the lateral membranes of adjacent cells (arrowheads). Scale bars: 1 μm (A-B); 5 μm (C); 2 μm (D).

The paca placenta is of the syncytial haemomonochorial type. A single trophoblast layer can be identified between the blood in the maternal blood spaces of the labyrinth and the endothelium of the fetal capillaries (Figure 7A–B). This trophoblast is syncytial in nature without cell boundaries and with large nuclei, often in close proximity to one other. Microvilli project from the apical membrane into the maternal blood space. Caveolae are seen in the apical membrane and the syncytiotrophoblast contains coated vesicles and larger vacuoles. There are recesses in the basal membrane. However, we saw no evidence of transtrophoblastic channels. There is an abundant amount of rough endoplasmic reticulum, a Golgi apparatus, lysosomes and numerous mitochondria.

In the interlobular areas, the syncytiotrophoblast bordering the maternal blood spaces has numerous microvilli (Figure 7C). The cytoplasm has abundant rough endoplasmic reticulum, mitochondria and electron-dense droplets. Cytotrophoblast cells occur within the syncytium. Desmosomes are present between adjacent cytotrophoblast cells as well as between cytotrophoblasts and the overlying syncytium.

The cells of the parietal yolk sac endoderm form a columnar epithelium (Figure 7D). These cells are irregular in shape with basally situated nuclei. The apical membrane, which faces the uterine lumen, has microvilli and caveolae. The supranuclear cytoplasm contains vacuoles and vesicles, tubular mitochondria and rough endoplasmic reticulum. Desmosomes are found between the lateral membranes of adjacent cells.

### Subplacenta and junctional zone

**Figure 8:**
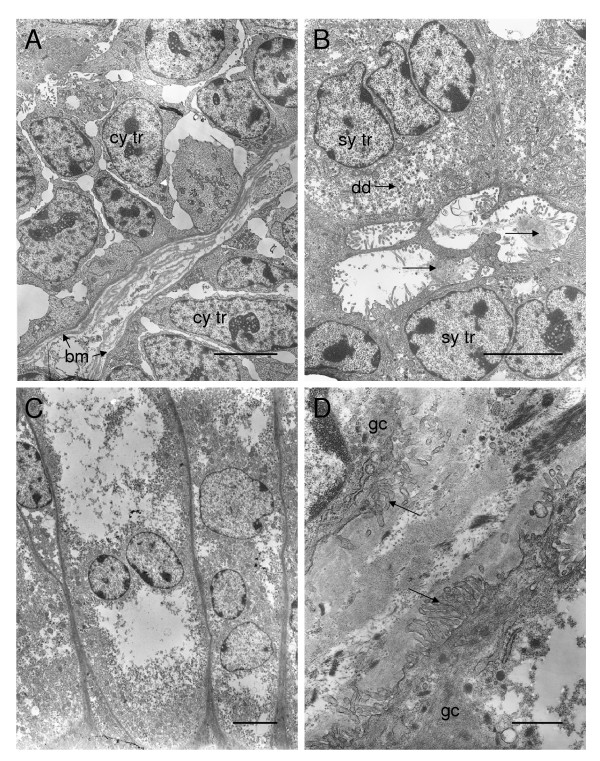
Ultrastructure of the subplacenta and junctional zone of the paca placenta. (A) The cytotrophoblast layer (cy tr) is multilaminar with dilated intercellular spaces. The cells are characterized by their large nuclei. They rest on a thin basement membrane (bm) which separates them from the fetal mesenchyme. (B) The syncytiotrophoblast (sy tr) contains electron-dense droplets (dd). Microvilli project from the syncytiotrophoblast into lacunae containing material of moderate electron density (arrows). (C) Multinucleated giant cells from the junctional zone. The cytoplasm has electron transparent areas, giving it a vacuolated appearance. (D) Two giant cells (gc) separated by intercellular matrix into which they send processes (arrows). Scale bars: 5 μm (A-C); 1 μm (D).

The cytotrophoblast layer is multilaminar with dilated intercellular spaces (Figure 8A). The cytoplasm of the syncytium has mitochondria, rough endoplasmic reticulum, numerous electron-dense granules (Figure 8B) and large accumulations of electron-dense material. The cytoplasm has electron transparent patches, which gives it a vacuolated appearance. There are lacunae within the syncytiotrophoblast, lined by microvilli and containing material of moderate electron density (Figure 8B). Desmosomes occur between adjacent cytotrophoblast cells and between the plasma membranes of these cells and that of the syncytial trophoblast.

In the junctional zone between the decidua and the lateral aspect of the subplacenta, there are nests of multinucleated giant cells (Figure 8C). Their morphology is variable. The cytoplasm has extensive electron transparent areas. The organelles tend to be confined to the perinuclear and marginal areas (Figure 8D) and include mitochondria, rough endoplasmic reticulum and electron-dense granules. The giant cells are separated by electron-dense material into which they send processes (Figure 8D).

### Yolk sac placenta

**Figure 9:**
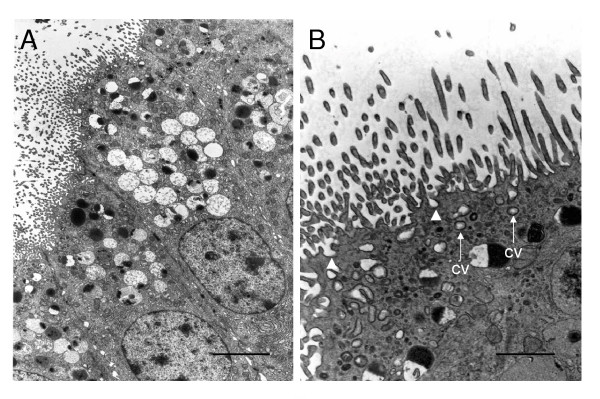
Ultrastructure of the inverted yolk sac placenta of the paca. (A) The apical surface of the endodermal cells bears numerous microvilli. The supranuclear cytoplasm contains many larger vesicles or vacuoles with a small amount of electron dense content. (B) Invaginations (arrowheads), small coated vesicles (cv) and tubules are present in the most apical regions of the cytoplasm. Scale bars: 4 μm (A); 1 μm (B).

The apical surface of the endoderm cells has numerous microvilli of relatively uniform length (Figure 9A). Small vesicles and tubules are present in the most apical regions of the cytoplasm (Figure 9B). The supranuclear cytoplasm contains a number of larger vesicles and vacuoles with a variable amount of electron-dense content. The perinuclear cytoplasm also houses a small Golgi complex. The cytoplasm has mitochondria and rough endoplasmic reticulum. Desmosomes and terminal bars are present between the lateral membranes of the cells.

## Discussion

As in other hystricomorph rodents [3,4], the placenta of paca consists of several lobes separated by interlobular trophoblast. The center of each lobe contains maternal arteries from which blood flows to the periphery through the trophoblastic channels of the labyrinth. In the fetal capillaries, blood flows from the periphery towards the center, allowing for countercurrent exchange [5,6]. The interlobular regions are made up of cords of syncytiotrophoblast, which define maternal blood spaces. We show here that each interlobular region drains several lobes.

In the labyrinth, the trophoblast is bathed directly by maternal blood and is separated from the fetal capillaries by a single layer of syncytiotrophoblast. Thus the placental barrier is syncytial haemomonochorial, as in the guinea pig [7-9], chinchilla [10], cane rat [11], degu [12] and rock cavy [13]. The apical membrane of this trophoblast, which is in contact with maternal blood, is well supplied with microvilli. There are recesses in the basal membrane and caveolae can occasionally be seen at the apical membrane. However, we did not observe transtrophoblastic channels such as those described in the degu [12].

In the interlobular region, the trophoblast contained mitochondria and rough endoplasmic reticulum. The surface in contact with maternal blood bore numerous microvilli. The interlobular trophoblast is the functional equivalent of the spongy zone of murid rodents [14]. In the guinea pig it has been identified as the principal site of progesterone synthesis [15]. In addition, it is thought to synthesize progesterone-binding protein [16], which is found in the plasma throughout gestation and is unique to hystricomorph rodents.

Much of the surface of the placental disk is covered by the endoderm of the parietal yolk sac. This epithelium is largely columnar and rests on Reichert's membrane. Beneath the membrane are trophoblast cells that differ from those in other regions of the placenta in their larger size, rounder form and vacuolated appearance. An appropriate designation for these cells is spongiotrophoblast. It has been shown in the guinea pig that maternal protein can cross the spongiotrophoblast and Reichert's membrane. It can then pass into the uterine lumen through the intercellular spaces between the endoderm cells, as they are not sealed by tight junctions [17]. In theory the protein could then be taken up by the visceral yolk sac, but whether maternal-fetal exchange occurs by this circuitous route is open to speculation.

The subplacenta is a unique feature of hystricomorph rodents [3]. Characteristic of the subplacenta of paca were the large intercellular spaces between the cytotrophoblasts and the lacunae within the syncytiotrophoblast. As in the guinea pig [18] and chinchilla [10], the lacunae in the syncytium were lined by microvilli and contained electron-dense material. It seems likely that the lacunae intercommunicate, but this requires further investigation. Wolfer and Kaufmann [19] suggested that the subplacenta might be a highly active area from a metabolic point of view. They pointed out that the structure had been carefully described, but that little was known about its function, except that it might have endocrine activity. Recently it was proposed that the subplacenta is an important source of invasive trophoblast in the guinea pig, chinchilla, capybara and degu [20].

We found multinucleated giant cells in the junctional zone between the subplacenta and decidua. Intriguingly, the cytoplasm of these cells contained electron-dense granules reminiscent of those found in the subplacental syncytium. The cytoplasm of the giant cells had areas of low electron density, a feature also shared by the subplacental syncytium. These cells were PAS-positive and may store glycogen or glycoprotein.

The placenta of paca is attached to the uterus by a prominent structure, formed largely of maternal tissue, that we have denoted the placental pedicle. It was first described by Strahl [21] and named by him the mesoplacenta. A similar structure occurs in the agouti [4], chinchilla [22] and nutria [5]. The equivalent formation in the capybara and guinea pig placenta is the placental stalk [23]. Trophoblast is found in the walls of the maternal vessels that pass through the pedicle to supply the placenta [4].

Like other hystricomorph rodents, paca has an inverted yolk sac placenta that persists until term. This visceral yolk sac displays folds and complex villi. The numerous digitiform projections are sometimes branched. They consist of a mesenchymal axis covered by a simple columnar epithelium of endodermal cells. The cells seem to have a high level of endocytotic activity. Similar characteristics are found in the yolk sac endoderm of the guinea pig [24], chinchilla [10] and rock cavy [13]. In the guinea pig it has been shown experimentally that immunoglobulin G is taken up from the uterine lumen to coated pits. The endocytotic vesicles thus formed are transported to the lateral membrane and empty into the intercellular spaces by exocytosis [25]. From here the immunoglobulins are presumed to reach fetal capillaries. Protein cannot move directly into the intercellular spaces because of the tight junctions near the apex of the cells [25]. In addition to this mechanism for conferring passive immunity to the fetus, there is nonspecific uptake of protein from the uterine lumen. Many endocytotic vesicles fuse with larger vacuoles that form part of the cell's lysosomal apparatus [26]. The protein they contain is thought to provide amino acids to the fetus. Given the similarity in ultrastructure, these mechanisms are likely to operate in the yolk sac placenta of paca.

In conclusion, the placenta of the paca conforms to patterns previously described for hystricomorph rodents [3,14]. Common features include the lobulation of the placenta and the presence of a subplacenta. At the ultrastructural level they comprise the haemomonochorial nature of the interhaemal barrier and the pinocytotic apparatus of the visceral yolk sac endoderm. The lobulated structure of the placenta allows for a larger exchange area and the development of precocial young [14].

Recently it was argued that more attention should be given to the hystricomorph rodents as models in human medicine. They bear a closer genetic similarity to humans than do murid rodents, such as the mouse and rat, because the latter have undergone a very high rate of gene mutation [27]. Since the paca carries a singleton fetus with a birth weight of 640–900 g, it deserves particular consideration as a potential model of fetal growth and development [28].
